# Measurement and removal of asbestos in residential dwellings to be demolished—urban transformation experience in Izmir, Turkey

**DOI:** 10.1007/s11356-023-31819-4

**Published:** 2024-01-10

**Authors:** Yılmaz Öğünç Tetik, İrem Bayram Zümrüt, Ayşe Gizem Çamurcu, Özge Akboğa Kale, Selim Baradan

**Affiliations:** 1 Department of Civil Engineering, Mugla Sitki Kocman University, Mugla, 48000 Turkey; 2 Department of Civil Engineering, Ege University, Izmir, 35040 Turkey; 3 Department of Civil Engineering, Izmir Democracy University, Izmir, 35140 Turkey

**Keywords:** Asbestos exposure, Asbestos-containing material, Removal process, Demolition, Air measurement

## Abstract

Asbestos has been used extensively in the construction industry for its superior insulation properties before its health hazards were discovered and its use eventually banned. It is likely that many residential buildings built before the 2000s in Turkey contain asbestos. Therefore, it is important to raise awareness of the potential danger of asbestos exposure during demolition work and to identify asbestos-containing materials and ensure their safe removal and disposal. This study is executed to determine the residential dwellings containing asbestos in Izmir, Turkey. The research included field studies to determine asbestos presence in the buildings that were damaged during the 2020 earthquake. Air measurements and bulk samples were taken from 50 buildings that would go through the demolition process. Eleven buildings were found to contain asbestos which corresponds to 22%. The detected asbestos type was 60% chrysotile (white asbestos). Results could be helpful for future demolition work, which are conducted in the same region that includes buildings with similar properties. Also, it is expected that the database created for this study could be useful in other studies in Turkey, where accurate statistical data related with asbestos measurements is essentially non-existent.

## Introduction

Asbestos was widely used in construction for its superior insulation and fireproofing properties (Craighead and Mossman 1982). Its application in various building materials, such as steel beams, columns, concrete, asphalt, vinyl, roof shingles, pipes, siding, wallboard, floor tiles, joint compounds, and adhesives, was common before its harmful health effects were discovered in the twentieth century. Due to its strength, asbestos was extensively employed for thermal insulation, including acoustical plaster and as a component in sprayed mixtures on ceilings and walls (Franzblau et al. 2020; Allen et al. 2018).

Inhaling asbestos fibers can lead to severe health conditions, including asbestosis, pleural disease, lung cancer, and mesothelioma, ultimately resulting in fatalities (Toyokuni 2019; O'Reilly et al. 2007). To address the lethal consequences of asbestos exposure, the European Union implemented Directive 1999/77/EC in 1999, prohibiting the use and marketing of asbestos in all member states. The directive took effect on January 1, 2005, marking the initiation of a global ban on asbestos, now observed in more than 60 countries. Despite these measures, asbestos-related deaths persist especially in nations with late bans due to their existing building stock containing higher amounts of asbestos than other countries. Turkey, having banned asbestos usage, extraction, and marketing in 2010, faces challenges due to the significant prior use of asbestos (Güneş, et al. 2017). This situation highlights ongoing concerns about asbestos exposure in regions with delayed regulatory actions. Today, damage to asbestos-containing materials (ACMs) still generates the release of asbestos fibers (Kim et al. 2015). The maintenance, repair, and dismantling operations and demolition of structures containing asbestos products continue to pose a hazard to workers and public health.

The Law of “Transformation of Areas Under Disaster Risks No. 6306” in Turkey, enacted in 2012, aims to address disaster-prone areas, particularly those at risk of earthquakes. The law focuses on principles and procedures for improving, demolishing, and renewing buildings in these high-risk zones to create suitable and safe living environments. Over the past decade, there has been a significant increase in demolition activities, with an estimated 7 million buildings expected to be replaced within 20 years (Bozdoğan 2020). In compliance with this law, nearly 22,000 buildings have been demolished, and approximately 60,000 more are anticipated to be demolished in the Izmir province alone (Kozanoğlu 2022). Besides, an earthquake magnitude of 6.9 struck Izmir in 2020, resulting in 23,985 damaged or collapsed buildings (DEMP 2021) and causing social and economic disruptions (Ekizler 2021).

During the urban renewal process and post-earthquake demolitions in Izmir, there is a high risk of encountering asbestos in buildings. Despite established regulations in Turkey regarding asbestos and construction/demolition work, many buildings are being demolished without adequate consideration for asbestos exposure. The current regulations lack detailed information, and pre-demolition processes are often not followed correctly by contractor firms, possibly due to misinformation or unawareness. Building owners typically claim no asbestos presence without conducting surveys leading to the potential exposure of workers and nearby individuals to hazardous asbestos levels. Studies have been conducted that addressed the issue by Kurt and Yıldırım (2016), Üzmezoğlu and Ocaktan (2017), and Taşbaşi et al. (2017). Also, a roadmap was proposed by Akboğa et al. (2017). However, challenges persist due to contractors and building owners being unwilling to cooperate in dealing with asbestos hazards, primarily attributed to a lack of awareness and lax enforcement of regulations.

**Fig. 1:**
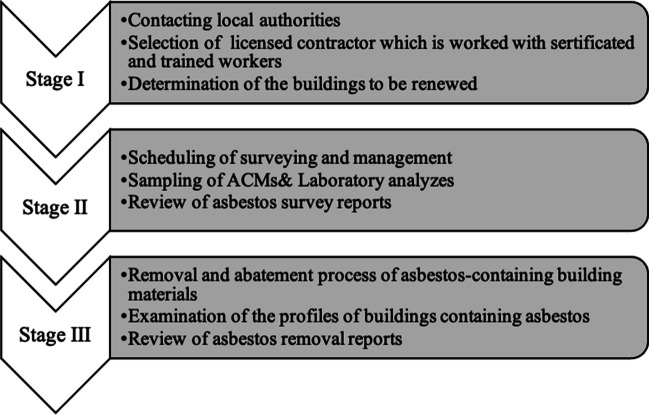
Flowchart of the methodology

This research aims to illuminate the asbestos situation in Izmir, Turkey, emphasizing the concern of demolition practices lacking proper asbestos control measures. Focused on the ongoing urban transformation in the city, the study examined buildings from diverse regions in Izmir to comprehend the broader landscape of asbestos-containing structures. The anticipated outcome is an increased risk of asbestos exposure, particularly during the urban renewal of older buildings constructed before the national asbestos usage ban. To assess the prevalence of ACMs in Izmir, three stages were followed. First, the local authorities were contacted, the company that would do the measurement and removal work was selected, and the buildings to be examined were selected (stage 1). After that, the sampling process of ACMs removal and abatement of asbestos-containing building materials was completed (stage 2–3) (Fig. 1).

## Methodology

### Asbestos sampling and analysis

In Turkey, there are regulations that contain specific guidance for working with asbestos, which are “Regulation on Health and Safety Precautions in Working with Asbestos (MLSS, 2013),” “Occupational Health and Safety Practice Guide in Working with Asbestos (MLSS, 2019)”, and “Regulation on Demolition of Buildings (MEU, 2021).” However, none of these offers detailed sampling and analysis methods. For this reason, based on the multiple asbestos removal contractor’s statement, it is seen that HSE 248 (HSE 2021) procedures are preferred for bulk and air sampling methods and HSE 264 (HSE 2012) survey strategies are applied, because HSE 248 offers details of bulk sampling methods for various ACMs in buildings such as floor tiles and pipe insulation. Respecting to instructions, in sampling of insulation board or tiles, if the area of the room is smaller than 25 m^2^, approximately 3–5 cm^2^ sample is taken. In sampling pipe or thermal insulation, one sample is taken every 3 m considering insulation layers.

In this study, fibers were identified by using polarized light microscopy (PLM) and phase-contrast microscopy (PCM) methods for the analysis processes. Due to asbestos analysis is still a new concept for Turkey, relatively more common and more practical methods were preferred (Thieves et al. 2022).

### Removal process

HSG 247 and 264 guidance were followed in this study for the removal process. Additionally, during the removal process, other regulations and procedures such as Guidance for Controlling Asbestos-containing Materials in Buildings (EPA 1985) and Work practices and engineering controls for Class I Asbestos Operations (OSHA 1994) were also considered.

**Fig. 2:**
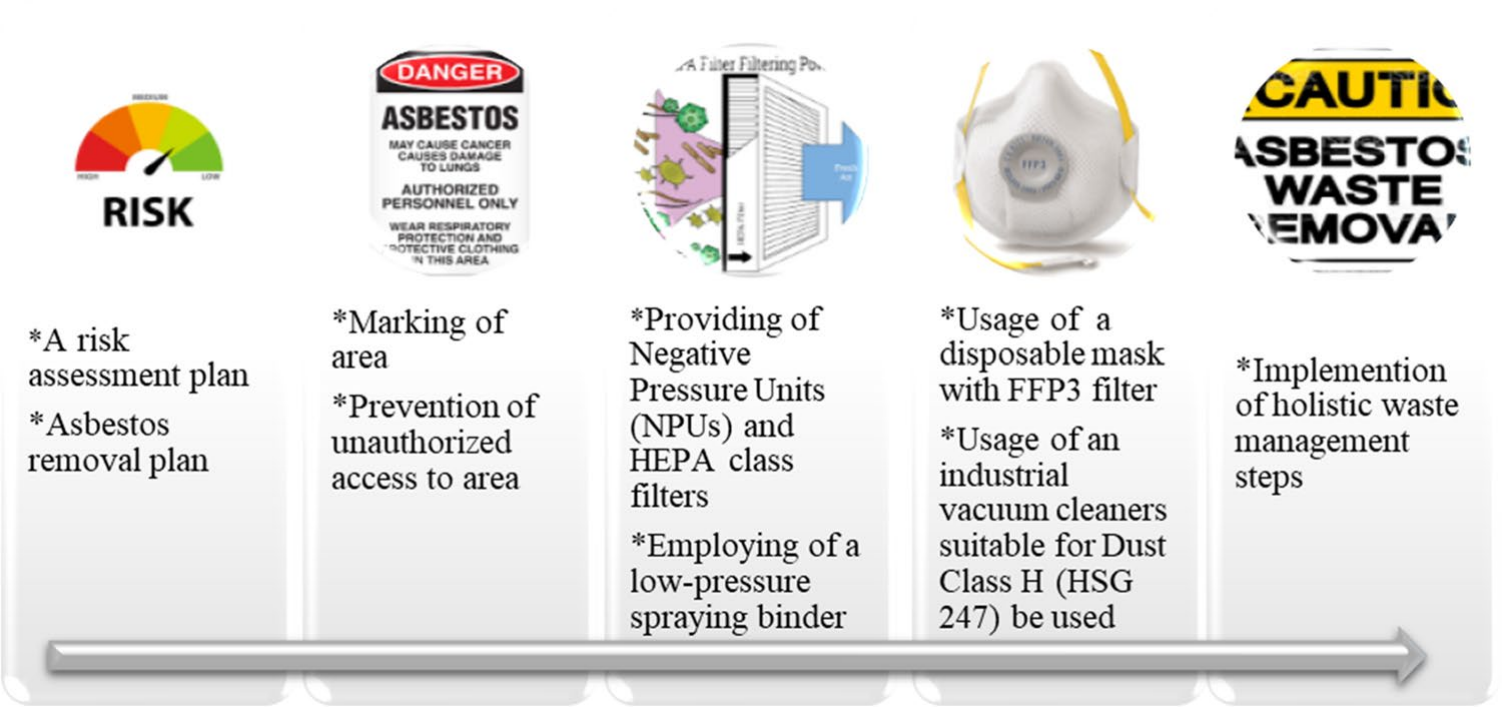
Summary of applied methodology fighting against asbestos

The removal methods were different according to the type or location of building materials. Nevertheless, the steps of method summarized in Fig. 2 were applied in each operation during the fieldwork, which is described in detail in the data acquisition process of the study. It is essential that these steps are followed in the removal of every building element where asbestos is determined.

## Case study

### Data acquisition process

**Fig. 3:**
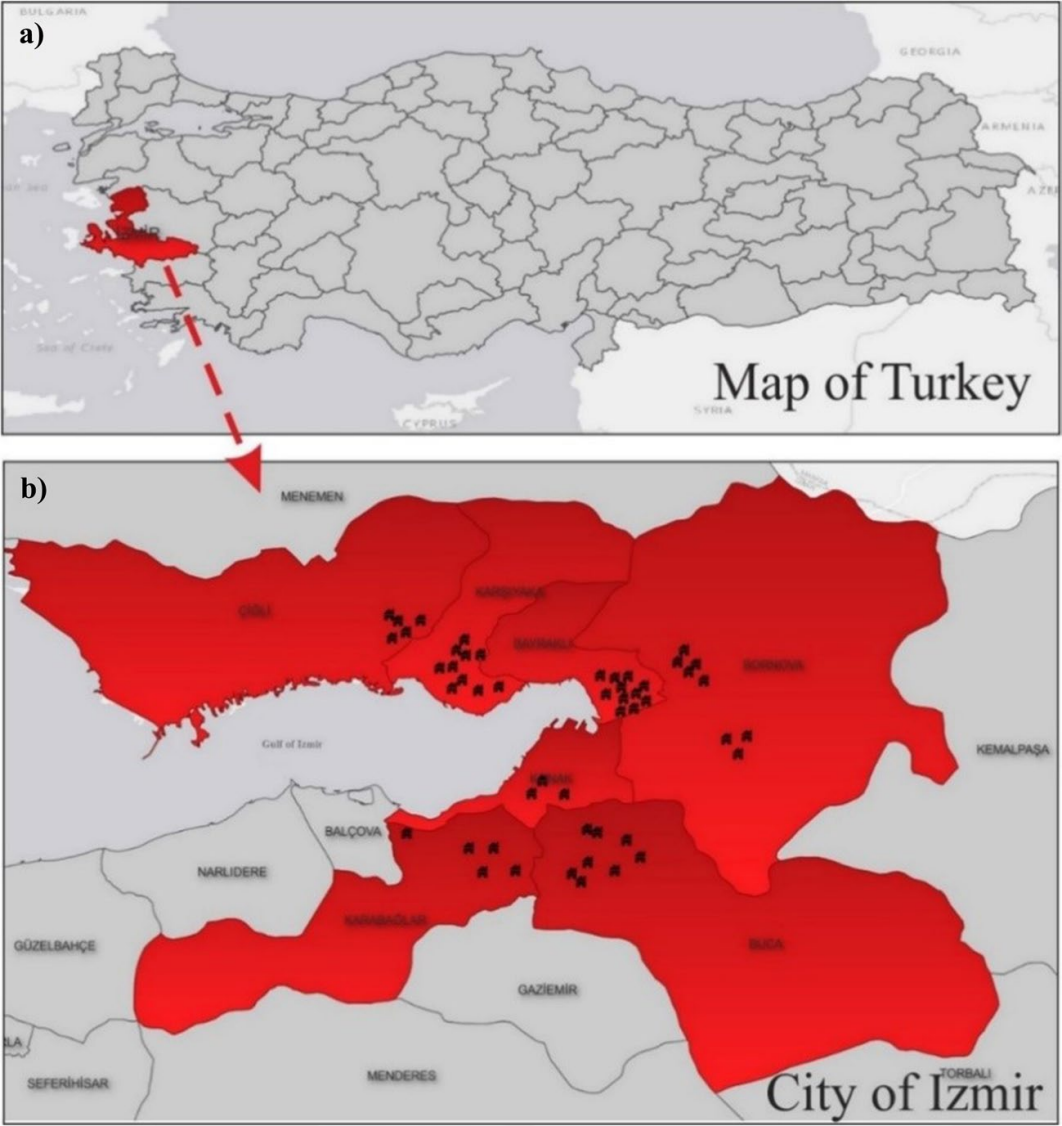
Location of Izmir (**a**) and distribution of targeted dwellings for sampling (**b**)

Izmir, located in the Aegean Region, is one of the most crowded cities with a population of 4,462,056 in Turkey (Fig. 3a). To determine the situation of asbestos-containing dwellings (ACDs) in Izmir, 50 buildings (total area—TA: 10,325 m^2^) were chosen. The construction dates of the dwellings vary between 1955 and 1998. The distribution of dwellings selected for sampling by seven districts which are 5 dwellings (TA: 740 m^2^) in Karabağlar, 8 dwellings (TA: 1050 m^2^) in Bornova, 5 dwellings (TA: 1155 m^2^) in Çiğli, 10 dwellings (TA: 1640 m^2^) in Karşıyaka, 8 dwellings (TA: 820 m^2^) in Buca, 3 dwellings (TA: 625 m^2^) in Konak, and 11 dwellings (TA: 4295 m^2^) in Bayraklı is given (Fig. 3b). These districts were intentionally chosen due to the urban renewal process rapidly ongoing and dwellings that are going to be demolished. The mean of the population of these seven districts is about 370.000 (TURKSTAT 2022).

**Fig. 4:**
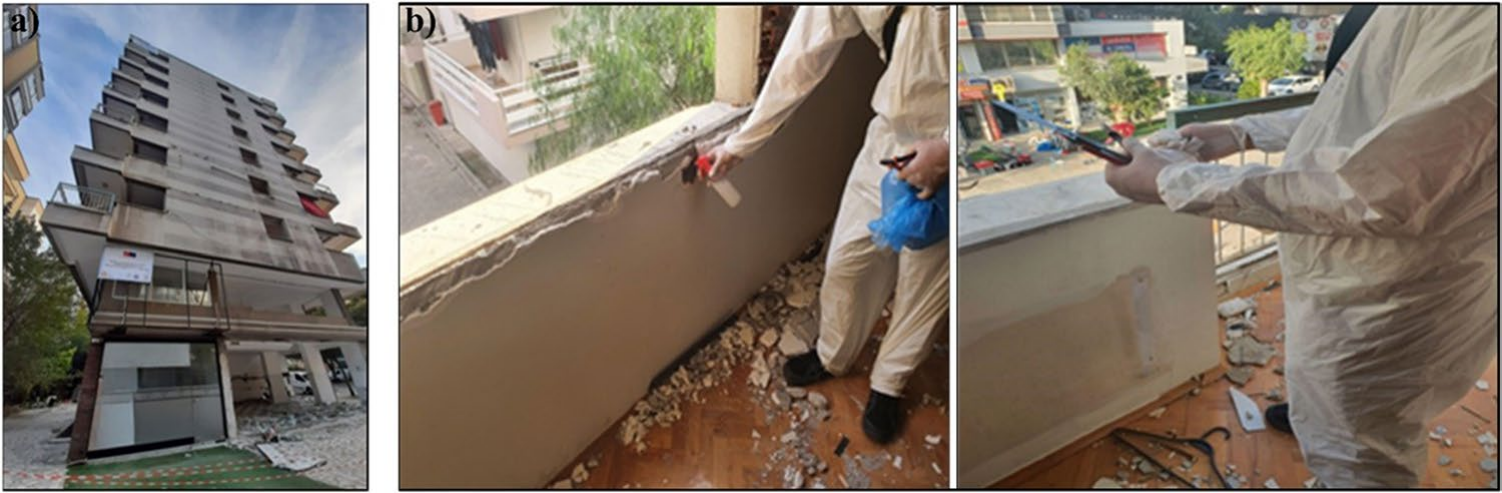
One of the target buildings (**a**) and sampling from wall covering (**b**)

**Fig. 5:**
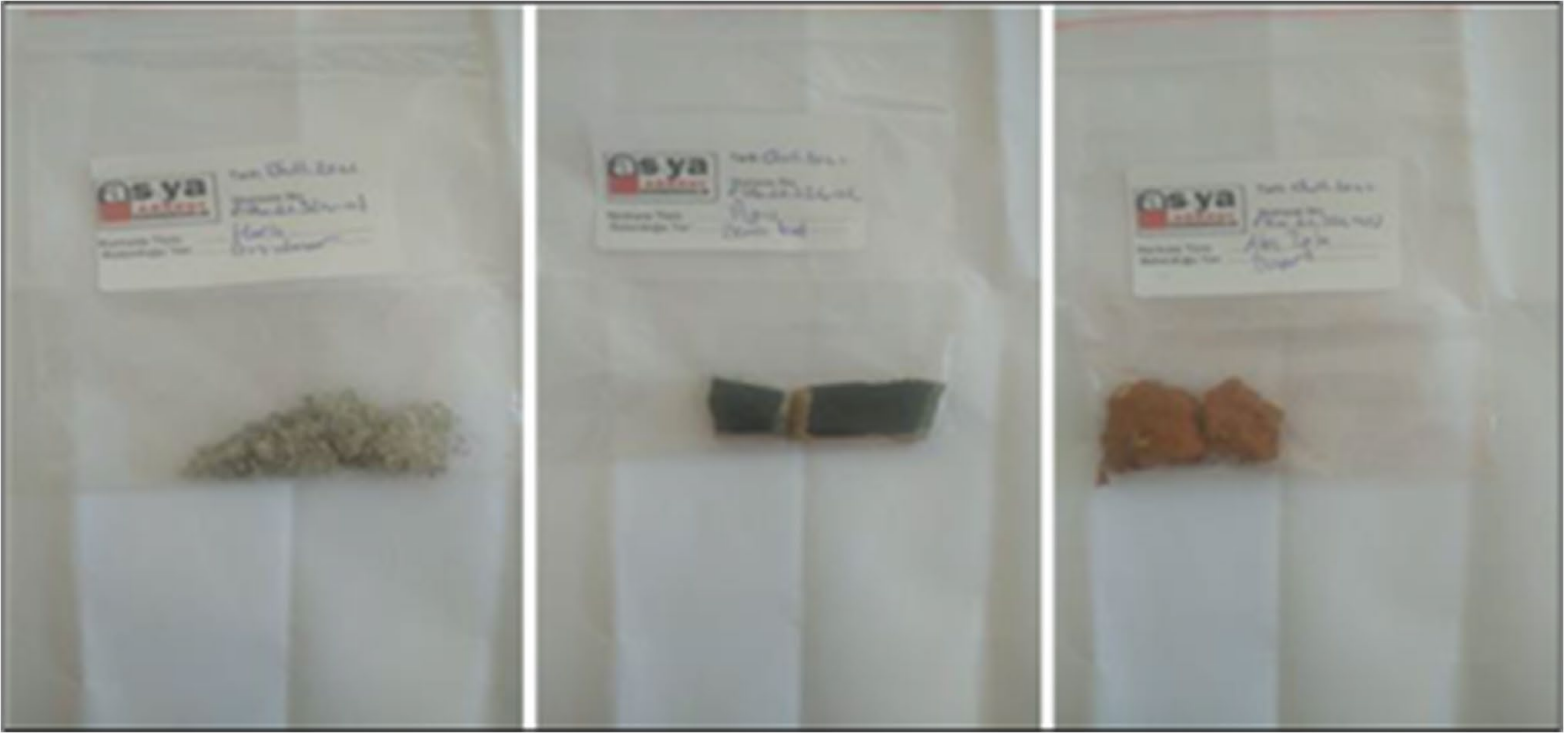
Packed and labeled bulk samples

To gather desired data, a licensed contractor was chosen which employs certificated asbestos removal experts and trained personnel in-house. It is known that when deciding on the number of samples to be taken for each location, an examination is carried out on the content of the material. Here, the final decision belongs to the team in sampling (HSG 248). However, MLSS suggested a minimum number of bulk samples depend on the number and area of the rooms (MLSS 2019). For this reason, according to specifications of residential dwellings (year of built, floor area in m^2^, number of story), it is decided that minimum 10 bulk samples taken from each building. Samples were mostly collected from floor tiles, wall coverings, roofing materials, insulation boards, decoration materials, etc. In total, 505 bulk samples were taken (Fig. 4a and b) and prepared for PLM analysis in an accredited laboratory (Fig. 5).

**Fig. 6:**
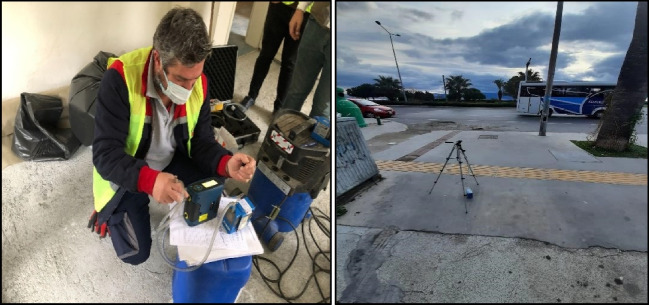
**a** Personal exposure gauges for air sampling. **b** Outdoor air measurement

Four air samples were taken from each building to determine the respirable asbestos fibers in the outdoor air. One sample was taken from outdoors during the removal process and one sample for personal exposure was taken instantaneously during removal and transportation for disposal. The outdoor air sampling is executed at a height of 1500 mm above ground level. The other two samples were collected as blind samples from the outdoor environment. The purpose of this procedure was to obtain reserve samples for use in case samples were damaged during the transportation and analysis process. In accordance with the NIOSH 7400 method, a utilized mixed cellulose ester membrane filter with a 25 mm diameter and pores measuring 0.8 μm were used in sampling. The preparation of personal exposure gauges is shown in Fig. 6a. Air samples taken from the outdoors are very close to the buildings, which detected asbestos in bulk materials (Fig. 6b). The reason for taking samples from the outdoor air is to find citizens’ exposure to fiber concentration. During the sampling process, the calibration of the pump’s flow rate was adjusted to 2–10 l/min.

**Fig. 7:**
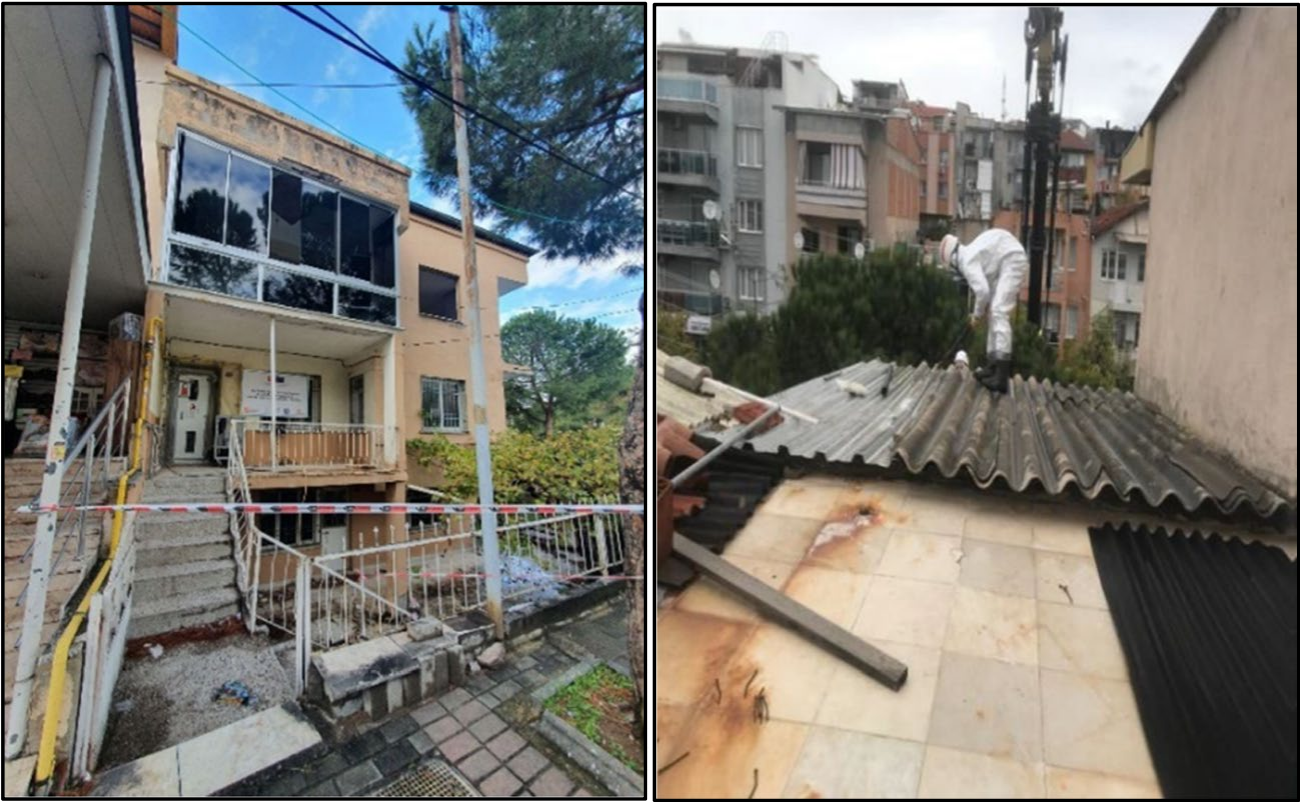
Asbestos removal process

**Fig. 8:**
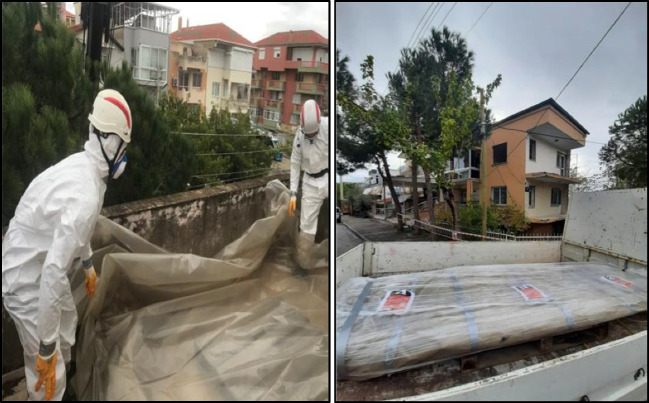
Packing the removed material and sending to the disposal facility

As part fieldwork of this study, the removal, transportation, and abatement process of a detected asbestos is depicted in Figs. 7 and 8. It is seen that roofing material is removed and packed by trained field personal. The forklift carried the package to the vehicle for transportation to the asbestos disposal facility.

### Findings

As a result of the measurements made for the study, a total of 505 bulk samples were taken from above described 50 buildings. As a result of the samples taken, asbestos was detected in a total of 11 buildings. All the buildings were residential dwellings with reinforced concrete structures. The detected asbestos type was 60% chrysotile (white asbestos). Chrysotile as a type of asbestos was identified with the various rates of asbestos content within the construction materials. Chrysotile was the most abundant variety in roofs and basements from all the studied buildings. It had been determined that the building material containing asbestos on the roofs was corrugated sheet, which is a roofing material made of asbestos and cement.

**Fig. 9:**
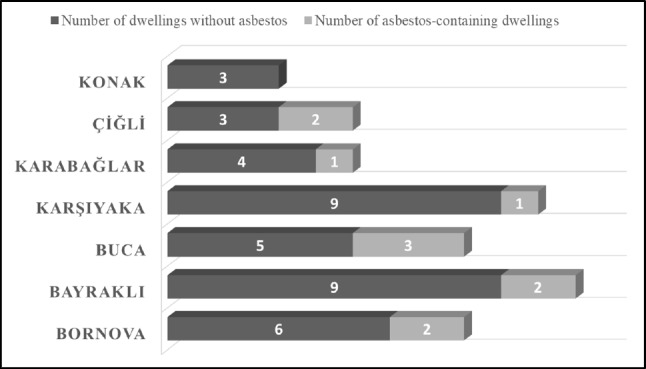
The distribution of ACDs in different districts of Izmir

ACDs were located in Buca (3), Bayraklı (2), Bornova (2), Çiğli (2), Karşıyaka (1), and Karabağlar (1). The distribution of the ACDs is shown in Fig. 9. The ratio of ACDs to the total number of buildings was obtained as 22% (11 ACDs in 50 buildings). The total area of ACDs was 2.112 m^2^ (20%). The ratio of ACM (%) of each building is given in Table 1. The minimum rate is calculated as 4% and the maximum rate of ACM in dwellings is 25%.

**Table 1:** The dwellings with ACMs and amount of removed ACMs

No	District	Construction date	Floor area (m^2^)	Number of floors	Location of ACM	Area of ACMs (m^2^)	ACM ratio (%)	Amount of ACMs removed (kg)
B-01	Karabağlar	1995	300	5	Roofing	40	13	250
B-02	Bornova	1995	100	3	Roofing	15	15	300
B-03	Çiğli	1990	105	2	Basement	8	8	100
B-04	Bornova	1980	131	2	Roofing	20	15	250
B-05	Karşıyaka	1973	215	6	Basement and roofing	30	14	400
B-06	Buca	1991	90	3	Roofing	30	33	250
B-07	Buca	1996	120	3	Roofing	30	25	250
B-08	Buca	1990	105	2	Roofing	10	10	100
B-09	Çiğli	1995	250	5	Garden	10	4	100
B-10	Bayraklı	1997	498	8	Roofing	25	5	250
B-11	Bayraklı	1997	198	10	Roofing	25	13	250

The districts of dwellings, year of construction, approximate floor areas, number of floors, location of detected ACMs, and calculated total area of ACMs which referred to overall asbestos surfaces (m^2^) were given in Table 1 with generated codes.

For the asbestos exposure assessment, two distinct metrics were employed in the study as air concentration and TWA (time-weighted average) value. Air concentration pertains to the quantifiable quantity or density of asbestos fibers present in the outdoor air of the measured while dismantling operations.

However, the TWA value constitutes a metric that portrays the average asbestos exposure experienced by person who is operating the dismantling operations. The sampling value is obtained from the worker by putting a device on him. The TWA value is calculated by using Eq. 1 which determines the mean concentration of asbestos fibers to which an individual is exposed during the workday, considering variations in exposure levels that may occur throughout the day. Both values are measured at the same time. In Eq. 1, *n* depicts the total number of samples.

1 $$TWA=\frac{sample\;1 \left(\frac{f}{{cm}^{3}}\right)\times estimated\;duration\;1 \left({\text{min}}\right)+ \dots \dots \dots ..\dots .+ sample\;n \left(\frac{f}{{cm}^{3}}\right)\times\;estimated\;duration\;(n) \left({\text{min}}\right)}{480 \left({\text{min}}\right)}$$

In Table 2, the air concentrations in the ACMs buildings and the calculated TWA are given.

**Table 2:** The air sampling results

No	Temperature (°C)	Pressure (hPa)	Sampled outdoor air volume (liters)	Sampled air volume for personal measurements (liters)	Fiber concentration in the air (fibers/cm^3^)	Personal 8 h (TWA) (fibers/cm^3^)
B-01	15	1017	480	240	0.0121	0.0097
B-02	17	999	800	199	0.0066	0.0188
B-03	17	1018	400	210	0.0121	0.0097
B-04	16	1016	380	256	0.0108	0.0141
B-05	15	1017	300	195	0.0185	0.0192
B-06	16	1018	260	182	0.0242	0.0152
B-07	14	1015	360	261	0.0174	0.0134
B-08	13	1013	660	495	0.0102	0.0053
B-09	15	1018	400	210	0.0121	0.0097
B-10	15	1017	320	203	0.0136	0.0125
B-11	16	1016	440	290	0.0099	0.0104

## Discussion

The amount of removed asbestos and results of air measurements (outdoor air and personal samples) were given in Table 1 and 2, respectively. Comparisons can be drawn with other studies on concentrations of asbestos-containing materials (ACMs) in demolished dwellings. Franzblau et al. (2020) sampled 605 dwellings in Detroit, USA, finding that 95% of demolished dwellings contained one or more types of ACMs. The most common materials with asbestos were flooring and roofing, present in about half of the sampled dwellings, along with siding and duct insulation, found in about one-third of the dwellings. Chrysotile asbestos was identified with varying rates in roof and basement materials, with duct insulation containing the highest fraction. Almost 99% of dwellings with asbestos had chrysotile content. Zhang et al. (2021) investigated 790 buildings in Busan, South Korea, and detected chrysotile and amosite types of asbestos in 34.3% of these buildings. The study also assessed the risk posed by the identified ACMs using four international methods, noting a high percentage of detected ACMs in roofing slates and gypsum cement boards (GBCs) in ceilings.

Air measurements from each building, as detailed in Table 2, indicate that the concentration of asbestos in the air, to which workers were exposed, remained below the permissible exposure limit (PEL) of 0.1 fiber/cm^3^ as an 8-h TWA (WHO 1998). In the existing literature, Wilmoth et al. (1994) found that workers’ exposure is less than 0.033 fibers/cm^3^ according to calculated TWA values. Perkins et al. (2007) reveal that workers exposed maximum 0.03 fibers/cm^3^ during the building demolition process. Phanprasit et al. (2012) found that asbestos fiber concentration in the air is tolerable for workers who are exposed during cutting asbestos cement roof sheets with either motor (0.13 fibers/cm^3^) or hand saw (0.01 fibers/cm^3^). Kakooei and Normohammadi (2014) measured personal exposure of asbestos fiber levels as a range from 0.01 to 0.15 fibers/cm^3^.

The air concentrations and personal exposure (TWA) values are significantly below the PEL in our study. This can be attributed to the fact that these measurements were taken during the disassembly process, which spanned 6–7 h and involved the implementation of rigorous safety measures. These precautions included: (1) minimizing dust by applying asbestos binder solution (liquid which binds the asbestos fibers), (2) dismantling ACMs without breakage, and (3) employing vacuum cleaners. Another reason for the results of the asbestos fibers in the outdoor air and personal exposure is close, because asbestos was detected only in one material that in ACDs. Also, there are no other ACM-containing buildings near the determined buildings which are going to remove asbestos.

In this study, fiber concentrations in outdoor air were measured as minimum 0.0066 PCM fibers/cm^3^ and maximum 0.0242 PCM fibers/cm^3^ in the environment surrounded by buildings where ACMs were detected. It is determined that the reference value of standard asbestos concentration has differences (Moteallemi et al. 2020). The outdoor air is equal to 0.00005 PCM fibers/cm^3^ (0.0022 SEM fibers/cm^3^) according to the World Health Organization (Khadem et al. 2018). The Victorian Asbestos Eradication Agency stated that air usually contains between 0.00001 and 0.0002 fibers/cm^3^ (VAEA 2023). This is because the fiber concentration in the air can vary widely depending on the location, characteristics of building materials, and demolition methods. Besides, the average temperature, relative humidity, and wind speed are other conditions that affect the respirable asbestos fibers in the air (Obminski 2020). In Korea, airborne asbestos was detected as 0.00062 TEM fibers/cm^3^ for urban areas (Lim et al. 2004). It is also seen that airborne asbestos fiber concentrations were measured 0.0059 PCM fibers/cm^3^ in Canada (Bourgault et al. 2014). Gualtieri et al. (2009) reported that asbestos fiber concentration was measured 0.00056 SEM fibers/cm^3^ in Italy which regard as quite high. Kakooei et al. (2013) found that 0.00118 PCM fibers/cm^3^ (0.0072 SEM fibers/cm^3^) asbestos in air of outdoor living areas in Iran. Besides, 0.0019 PCM fibers/cm^3^ (0.0072 SEM fibers/cm^3^), 0.00111 ± 0.00025 PCM fibers/cm^3^ (0.01221 ± 0.00252 SEM fibers/cm^3^), and 0.00184 PCM fibers/cm^3^ (0.01816 SEM fibers/cm^3^) airborne asbestos were reported for different regions in Iran (Taghizadeh et al. 2019; Fathi et al. 2017; Kermani et al. 2021). Also, Neitzel et al. (2020) reported for Detroit, USA, that the emissions of airborne asbestos during emergency demolition operations appear to be negligible for the determined limit of detection levels which are 0.00038 to 0.5 PCM fibers/cm^3^ and 0.000086 to 0.013 TEM fibers/cm^3^.

## Conclusion

Considering the existing building stock, it is known that ACMs are used in many buildings that were built in the past. The number of demolitions is continuously increasing as part of the national urban transformation process. Therefore, the risk of asbestos exposure is accordingly increasing.

This study appears to be the first to summarize the characteristics of asbestos in the residential dwellings in a large-scale urban transformation phase effort in Turkey. The ratio of ACMs in dwellings depicts the significancy of asbestos surveying and removal process before demolition works in the city of İzmir. In the scope of the study, 505 bulk samples were taken from different locations of 50 residential dwellings in Izmir, Turkey. Results indicate that 11 buildings contain asbestos which this rate corresponds to 22%. It is also concluded that the ratio of ACM in dwellings is between 4 and 25%. TWA measurements results showed that the concentration of asbestos that workers were exposed to did not exceed the PEL of 0.1 fiber/cm^3^. Also, respirable asbestos fiber concentration in the outdoor air is relatively low compared to other results in the literature.

Considering that asbestos is not banned all over the world and the production of chrysotile type asbestos continues in some countries, the harms of this substance, which is a global danger to human health, and methods of protection should continue to be examined in detail. It is expected that the database created for this study could be useful in other statistical studies involving Turkey, where accurate statistical data related to asbestos measurements is essentially non-existent. Furthermore, a similar study could be carried out in different locations and comparisons could be made with the findings of this study.

For future studies, it will be very beneficial to expand the research by analyzing the results with the TEM method, which is much better in terms of countable fibers by ability of magnification, the smallest fiber width that can be seen and identification of fiber types, and identification of asbestos type without a doubt. In addition, when the relevant sources are examined, it has been determined that the guides (HSE 247, 2006, HSE 248, HSE 264 etc.) that can be used are not fully translated into their native language. Such manuals need to be translated considering the dynamics of local parameters such as technological advancements, infrastructures, logistics, and qualified personnel.

## Data Availability

The data that support the findings of this study are available upon request from the authors.
